# Supplementary material for: Shape-memory effects in molecular crystals
Source: Nat Commun. 2019 Aug 19;10:3723. doi: 10.1038/s41467-019-11612-z (PMC6700106; doi:10.1038/s41467-019-11612-z)
Supplement: Supplementary file 1 — Supplementary Information [file 41467_2019_11612_MOESM1_ESM.pdf]

SUPPLEMENTARY INFORMATION

Shape-Memory Effect in Molecular Crystals

Ahmed, Karothu, et al.

Contents

Supplementary Notes……………………………………………………………3
Supplementary Tables …………………………………………………………. 4–7
Supplementary Figures ………………………………………………………8–34

Supplementary Notes

Supplementary Note 1. The elastic and plastic bending here should not be confused with the elastic and plastic regime of mechanical response of a crystal to tensile or compressive stress.

Supplementary Note 2. One would ideally expect that this would appear as terraces at the end of the crystal, however such structure has not been observed yet, perhaps due to the fact the layers are also compressed during bending and this compression, together with the relatively longer length of the straight section at both ends of the crystal, results in dissipation of the strain.

Supplementary Note 3. The curvature is defined as the reciprocal of the radius of the arc that best approximates the curved crystal.

Supplementary Tables

Supplementary Table 1. Unit cell parameters determined for a crystal that was contacted with a metal object (labels A–D correspond to the respective panels in Supplementary Figure 4)

	**A**	**B**	**C**		**D**
**Temperature / K**	**290**	**290**	**290**	**290**	**290**
**Polymorph**	**Form II**	**Form I**	**Form II**	**Form I**	**Form II**
Crystal system	Triclinic	Triclinic	Triclinic	Triclinic	Triclinic
Space group	$P\bar{1}$	$P\bar{1}$	$P\bar{1}$	$P\bar{1}$	$P\bar{1}$
a / Å	5.14	3.73	5.13	3.82	5.13
b / Å	5.52	6.49	5.39	6.54	5.39
c / Å	7.22	7.39	7.02	7.40	7.02
α / °	71.92	82.95	72.35	83.40	72.35
β / °	75.84	81.49	76.91	80.78	76.91
γ / °	87.18	89.23	87.82	89.43	87.82
Volume / Å^3	189	175	180	181	180

Supplementary Table 2. Unit cell parameters determined for a shape-restored crystal after it has been uniformly pressed with a metal plate (the locations a–d refer to Supplementary Figure 12)

	**Damaged form II**	**a**	**b**	**c**	**d**
**Temperature / K**	**290**	**362**	**362**	**362**	**362**
**Polymorph**	**Form II**	**Form I**	**Form I**	**Form I**	**Form I**
Crystal system	Triclinic	Triclinic	Triclinic	Triclinic	Triclinic
Space group	$P\bar{1}$	$P\bar{1}$	$P\bar{1}$	$P\bar{1}$	$P\bar{1}$
a / Å	5.02	3.77	3.70	3.72	3.72
b / Å	5.34	6.47	6.49	6.46	6.48
c / Å	6.99	7.38	7.33	7.38	7.36
α / °	72.01	82.94	83.85	83.31	83.35
β / °	76.01	81.55	80.37	80.45	80.03
γ / °	87.32	88.99	86.88	88.54	87.50
Volume / Å^3	173	177	173	174	174

Supplementary Table 3. Unit cell parameters determined for a shape-restored crystal after it has been heavily damaged by using a metal object (the locations a–c refer to Supplementary Figure 14)

	**Heavily damaged form II crystal**	**a**	**b**	**c**
**Temperature / K**	**290**	**360**	**360**	**360**
**Polymorph**	**Form II**	**Form I**	**Form I**	**Form I**
Crystal system	Triclinic	Triclinic	Triclinic	Triclinic
Space group	$P\bar{1}$	$P\bar{1}$	$P\bar{1}$	$P\bar{1}$
a / Å	4.97	3.93	3.80	3.81
b / Å	5.37	6.36	6.39	6.42
c / Å	6.94	7.35	7.40	7.38
α / °	72.02	84.58	83.42	83.05
β / °	75.85	79.65	80.89	81.08
γ / °	85.90	88.04	88.28	88.42
Volume / Å^3	171	180	176	177

Supplementary Table 4. Crystallographic data and refinement details of the two domains in a bent crystal of terephthalic acid refined from the convex (form II) and concave (form I) regions of a bent crystal

**Bent region**	**Convex**	**Concave**
**Temperature / K**	**290**	**290**
**Polymorph**	**Form II**	**Form I**
Formula weight	166.13	166.13
Crystal system	Triclinic	Triclinic
Space group	$P\bar{1}$	$P\bar{1}$
a / Å	4.97280(10)	3.66490(10)
b / Å	5.30140(10)	6.3683(2)
c / Å	6.9746(2)	7.4304(2)
α / º	72.653(2)	83.723(2)
β / º	74.880(2)	79.758(3)
γ / º	86.245(2)	87.345(3)
Volume / Å^3	169.416(7)	169.569(9)
Z	2	2
Density / (g cm^{-3})	1.628	1.627
μ / mm^{-1}	0.125	0.125
F_{000}	86	86
h_{min}, h_{max}	–5, 4	–4, 4
k_{min}, k_{max}	–5, 5	–7, 7
l_{min}, l_{max}	–6, 6	–8, 8
No. of measured reflections	648	1423
No. of unique reflections	386	460
No. of reflections used	348	453
R_{all}, R_{obs}	0.09, 0.08	0.100, 0.099
$wR_{2,all}$, $wR_{2,obs}$	0.238, 0.234	0.343, 0.342
$\Delta\rho_{min,max}$ / (e Å^{-3})	-0.506, 0.450	-0.611, 0.812
GooF	1.179	1.290
CCDC No.	1854403	1854404

Supplementary Table 5. Unit cell parameters determined from the straight and bent sections from the same bent crystal of form II terephthalic acid

	**Bent form II**	**Straight form II**
**Temperature / K**	**290**	**290**
**Polymorph**	**Form II**	**Form II**
Crystal system	Triclinic	Triclinic
Space group	$P\bar{1}$	$P\bar{1}$
a / Å	5.03	5.11
b / Å	5.38	5.33
c / Å	7.00	6.99
α / º	72.28	72.29
β / º	75.84	76.04
γ / º	87.14	88.11
Volume / Å^3	175	176

Supplementary Figures

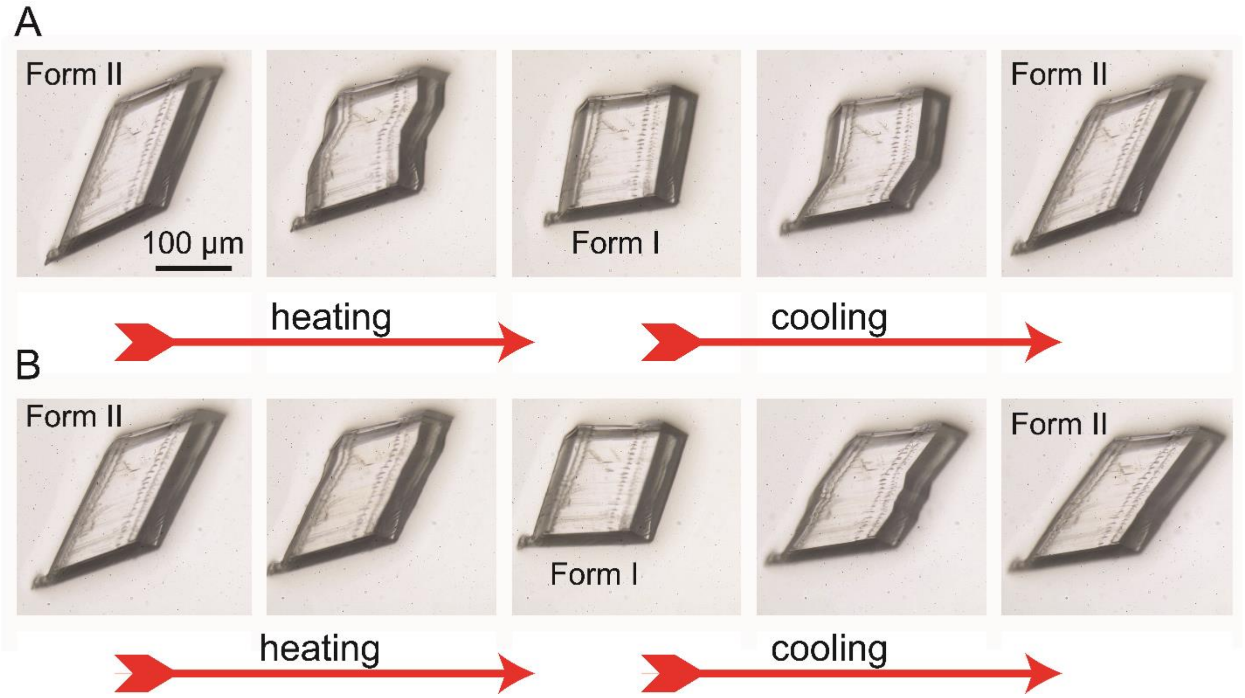

Supplementary Figure 1│A single crystal of form II terephthalic acid, consecutively taken twice over the phase transition to form I by heating and cooling. Panels A and B show the first and the second heating and cooling cycle between 296 K and 360 K. The crystal does not show any visible deterioration.

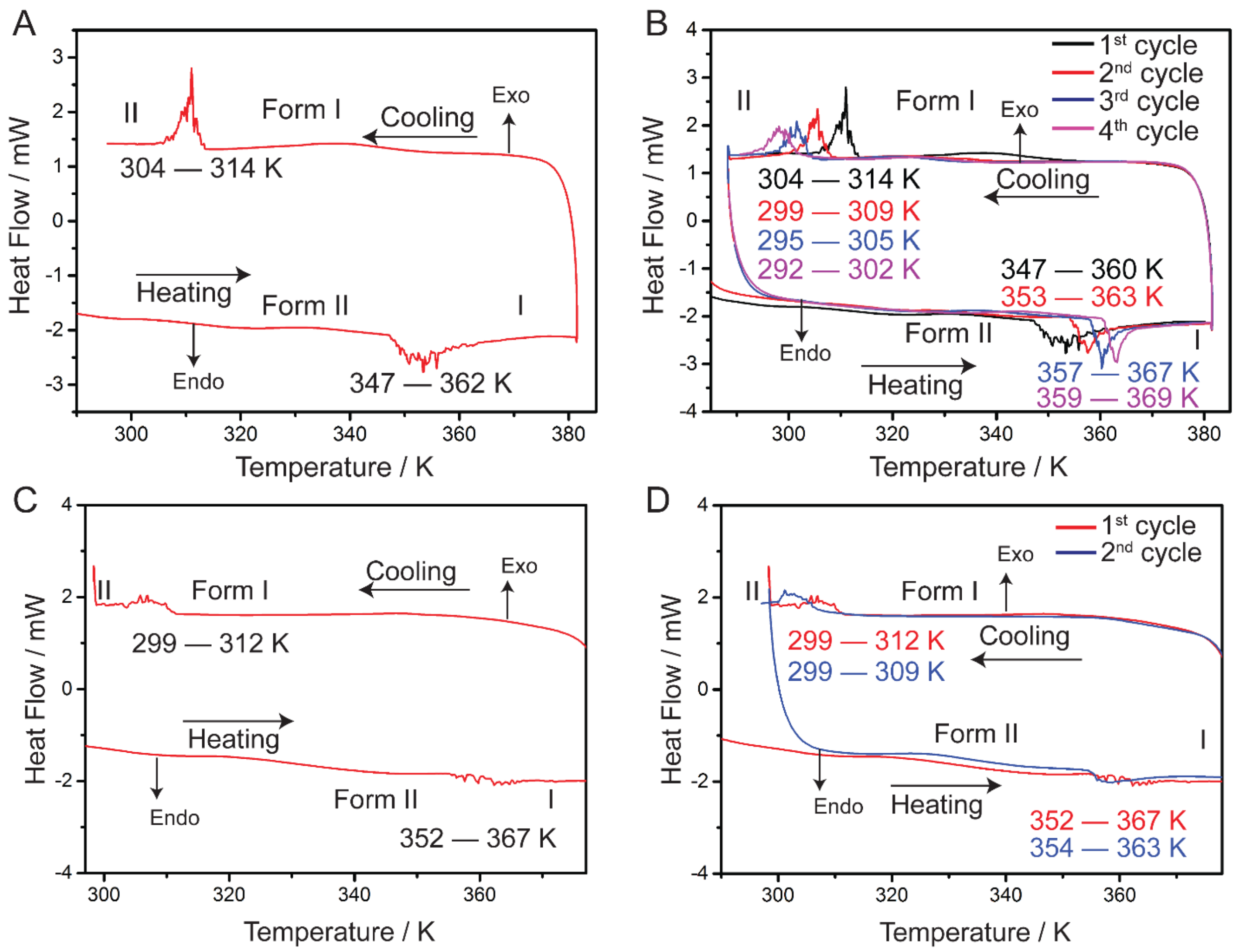

Supplementary Figure 2│ Differential Scanning Calorimetry (DSC) of as-obtained (A, B) and lightly ground (C,D) crystals of form II terephthalic acid (TA). (A) DSC profile recorded by heating and cooling of crystals of TA over the temperature of phase transition. (B) DSC profile recorded over four consecutive thermal cycles. Note the slight offset in the range of transition temperatures between the consecutive cycles. (C) DSC profile of lightly ground crystals of TA. Note that similar to other thermosalient transitions (*J. Am. Chem. Soc*. 2013, 135, 12241), the peak intensity is significantly alleviated compared to the non-ground crystals. (D) Two thermal cycles in the DSC of lightly ground crystals. The heating and cooling rates in all experiments were 10 K min^{-1}.

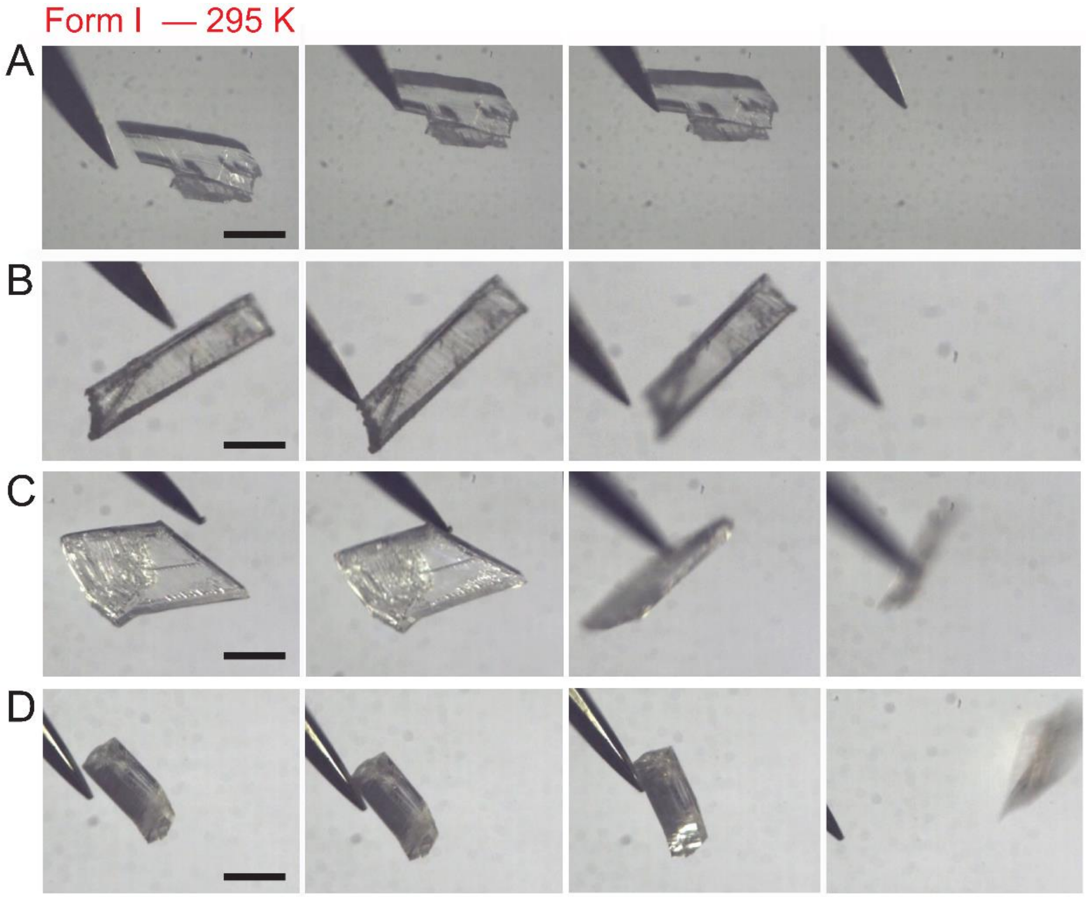

Supplementary Figure 3 | Mechanosalient effect of crystals of form I terephthalic acid captured by high-speed camera at a rate of 1500 frames per second. Initially, crystals of form II were heated and transformed to form I. After cooling, most of the crystals converted to form II, however some of the crystals remained in form I, which is metastable at room temperature and ambient pressure. These crystals jump when they are lightly contacted with a metal needle. Scale bars: (A) 600 µm; (B) 500 µm; (C) 400 µm; (D) 300 µm.

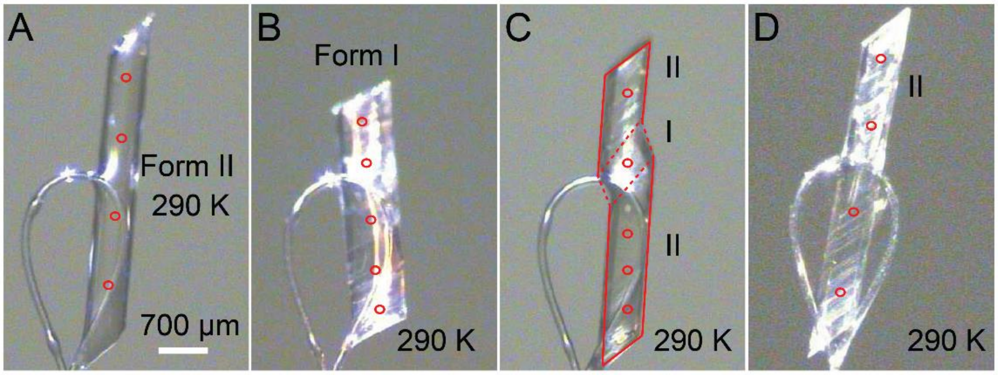

Supplementary Figure 4 │ A single crystal used for *in situ* X-ray diffraction analysis of a mechanically stimulated phase transition. A crystal of form II terephthalic acid was covered in Paratone to slow down the transition and mounted on the diffractometer head. The crystal was taken over the transition between form II and form I several times by repeated heating-cooling cycles, and was slowly cooled to room temperature, where form I is metastable. Once the crystal in the metastable form I is lightly contacted with a metal needle, it starts to transform to form II. The unit cell was determined at different positions of the crystal before and during the transition to confirm its phase identity. After 15 minutes, the crystal was completely transformed to form II, and diffraction data were collected again. The unit cell parameters are available from Supplementary Table 1.

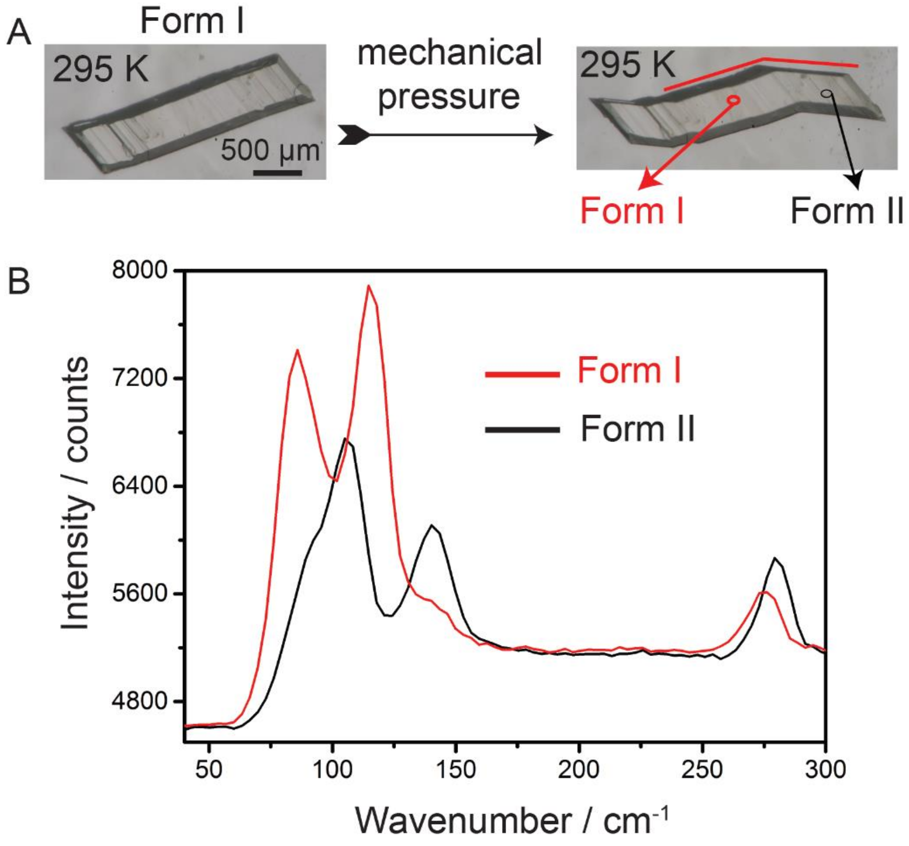

Supplementary Figure 5│ Low-frequency region in the Raman spectra of partially transformed crystal of terephthalic acid. (A) A crystal of form I was contacted with a metal object whereupon it was partially transformed to form II. The domains of the two phases are highlighted on the image. (B) Raman spectra recorded from the two domains of the partially transformed crystal showing the phase difference.

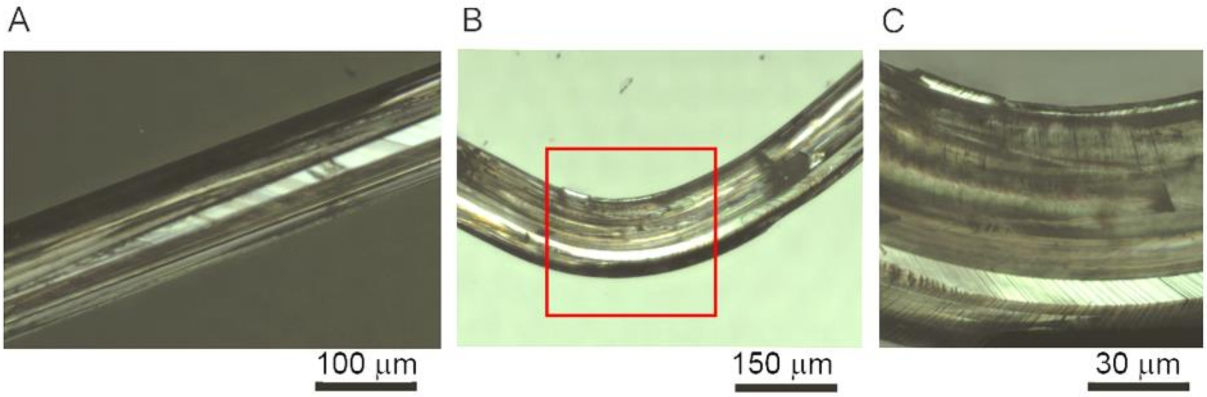

Supplementary Figure 6│Evolution of striations on the crystal surface upon bending. (A) Optical image of a straight crystal of form II. (B) The same crystal after three-point bending. (C) The bent region marked with red square in panel B shown at higher magnification.

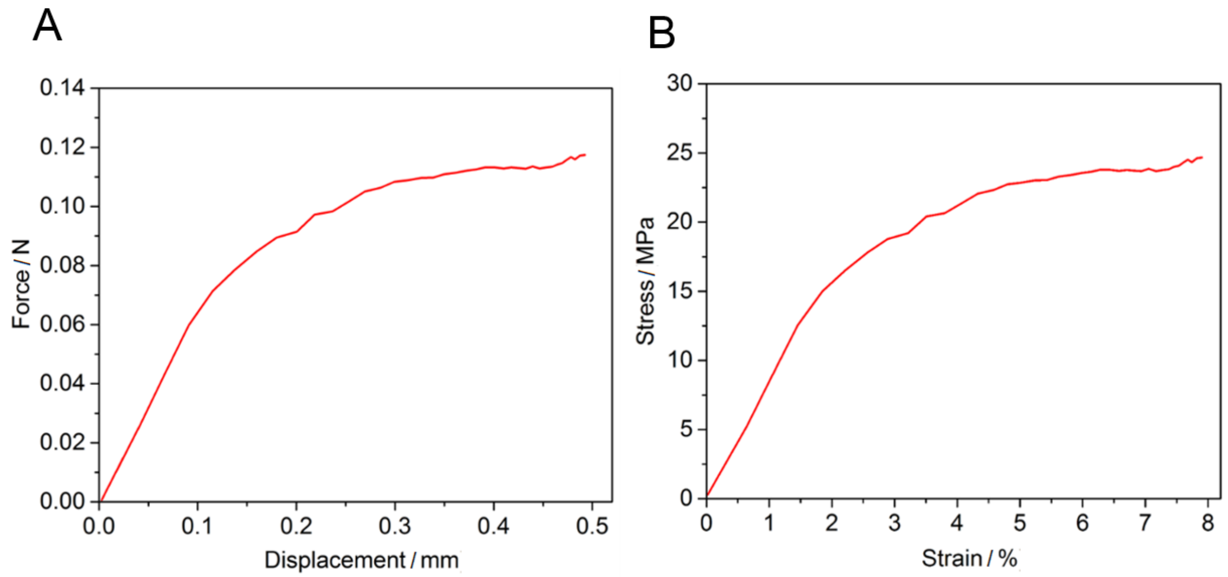

Supplementary Figure 7 | Mechanical properties of a crystal of form II terephthalic acid. Panels A and B show the force-displacement and the stress-strain curves, respectively. The Young's modulus calculated from the stress-strain curve is 0.81 ± 0.05 GPa. Note that this value is very different from the one obtained by nanoindentation (6.2 ± 0.7 GPa; for details, see the main text).

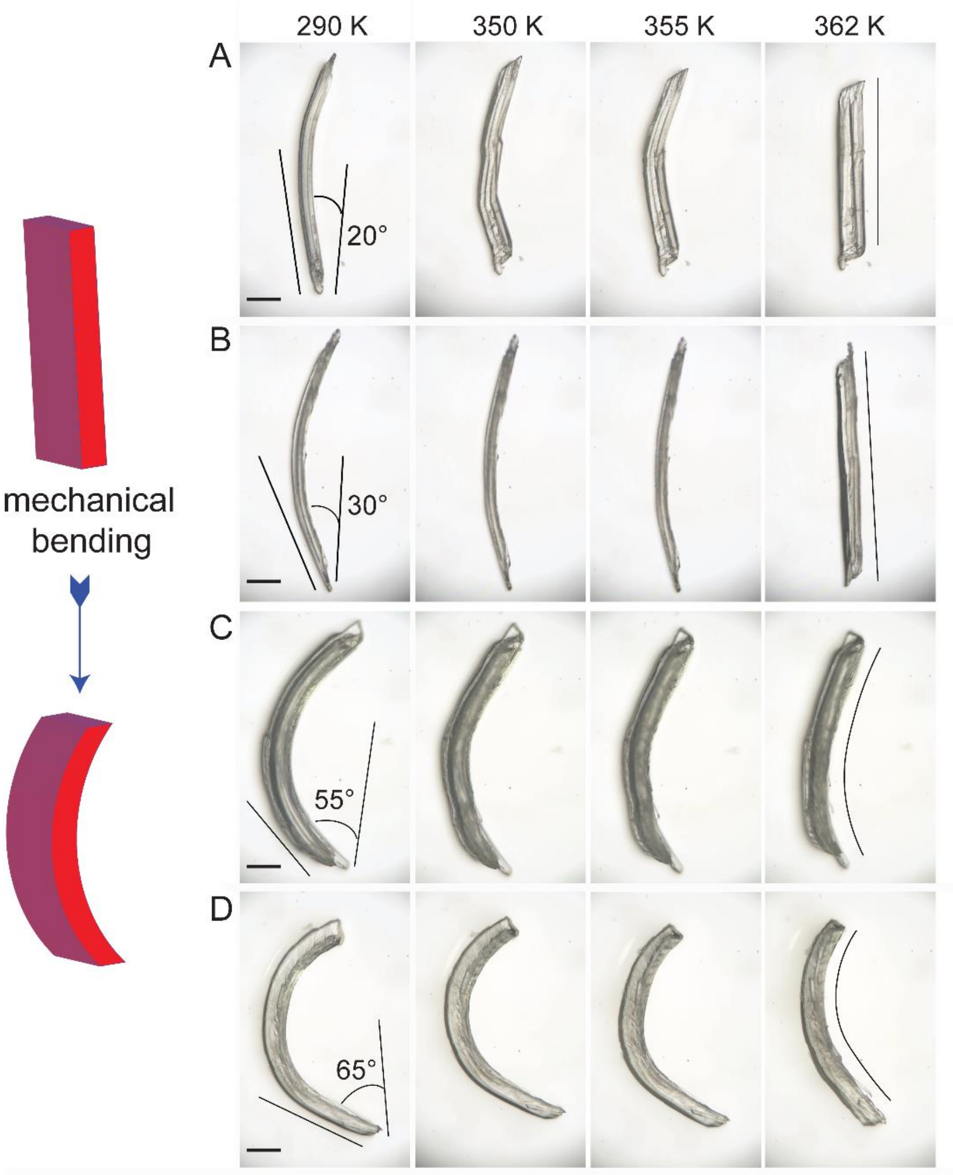

Supplementary Figure 8│ Shape memory effects of crystals of form II terephthalic acid that were bent to various extent. The (initially straight) crystals of form II were mechanically bent to approximate angles of 20° (A), 30° (B), 55° (C) and 65° (D), as shown in the optical images. These bent crystals were then taken over the phase transition to form I by heating from 300 K to 362 K. Some of the thinner crystals having smaller bending angle showed complete shape restoration (panels A and B) while thicker

crystals having more acute bending angle showed partial shape restoration (panels C and D). Scale bars: (A) 500 µm, (B) 500 µm, (C) 400 µm, (D) 600 µm.

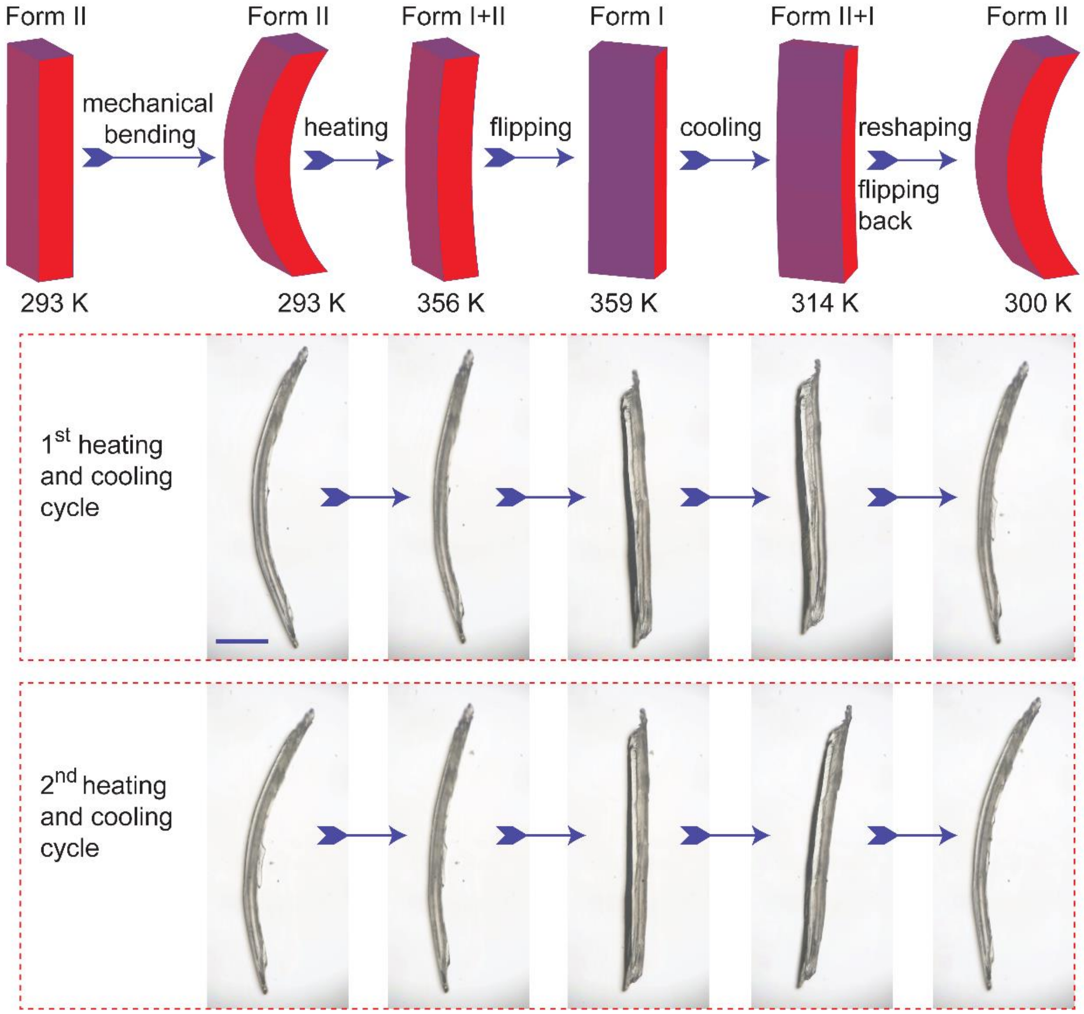

Supplementary Figure 9│ A bent crystal of form II terephthalic acid, consecutively taken twice over the phase transition to form I. During the heating from 293 to 359 K the bent crystal recovered its straight shape. After cooling to room temperature it returned to its initial bent shape. The crystal does not show any visible deterioration compared to its initial appearance. Scale bar, 600 µm.

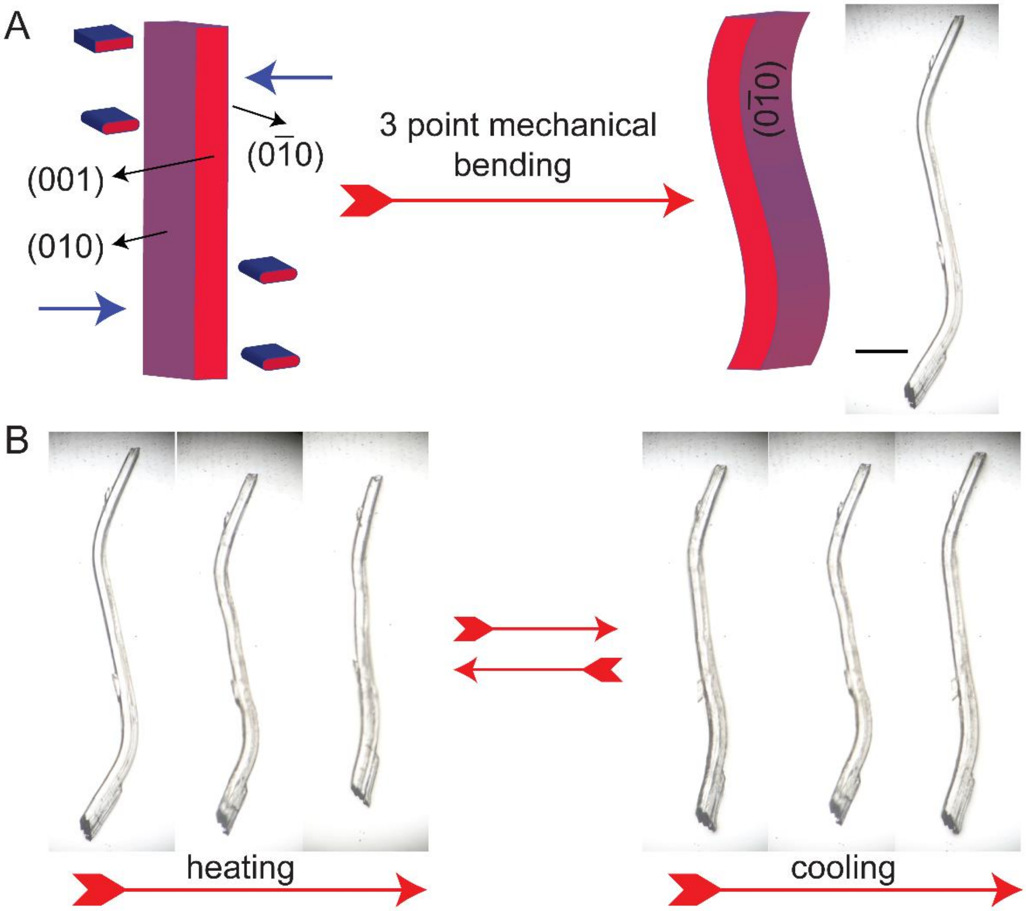

Supplementary Figure 10│ Deformation of a crystal of form II terephthalic acid upon application of force on oposite faces of the crystal. (A) Schematic of the three-point bending of the crystal performed by applying force on its (010) and ($0\bar{1}0$) faces. (B) Shape-memory behavior of an S-shaped crystal upon heating and cooling. Scale bar: 800 µm.

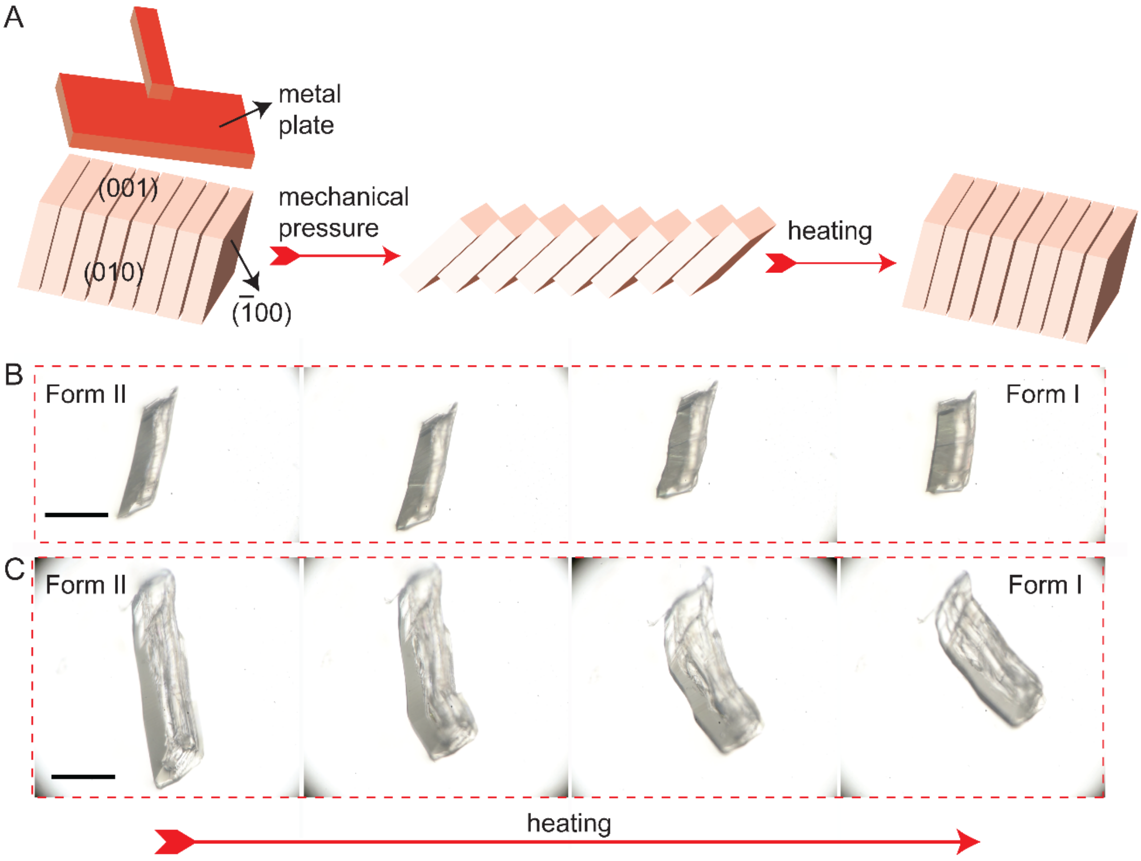

Supplementary Figure 11│Heat-induced restoration of macroscopic integrity of cracked crystals of form II TA which were pressed uniformly on their (001) face. (A) Schematic of the restoration mechanism. (B,C) Shape restoration of lightly compressed (B) and heavily compressed (C) crystals. Scale bars: (B) 300 µm; (C) 800 µm.

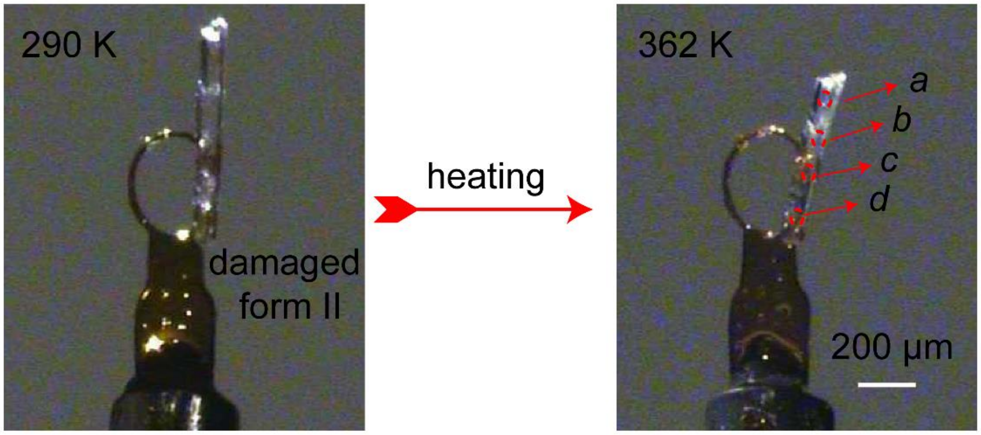

Supplementary Figure 12│ Image of a crystal of TA used in the unit cell measurements of the self-restoration effect of pressed crystals. Initially, a crystal of form II was damaged by applying force with a metal plate on its (001) face and mounted on the loop. The damaged crystal was taken over the phase transition temperature and its unit cell was determined at four locations (marked a, b, c and d). The unit cell corresponds to that of form I. The unit cell corresponds to that of form I. The unit cell details are available from Supplementary Table 2.

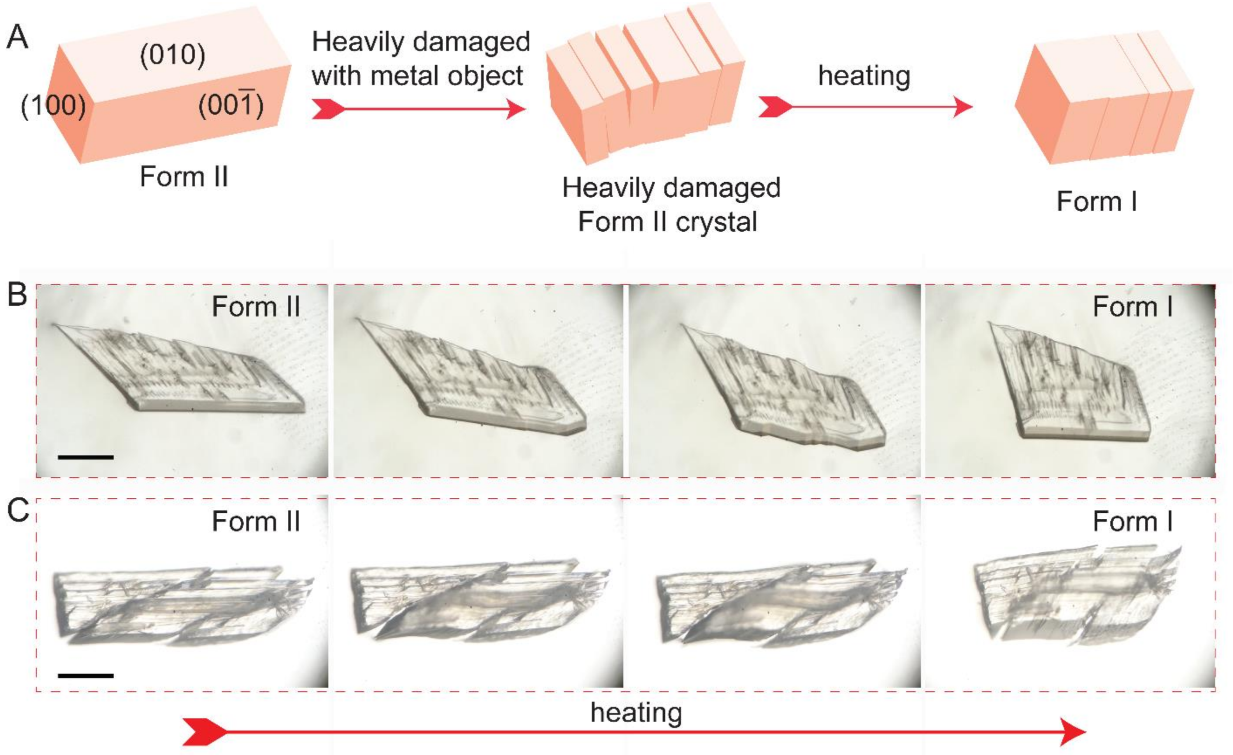

Supplementary Figure 13│ Propensity for restoration, after heating, of integrity of crystals of form II TA that were damaged with a metal object on their (010) face. (A) Schematic of the shape restoration mechanism of a crystal that has been damaged by applying relatively uniform pressure across its surface by using a metal object. (B,C) Shape restoration of slightly (B) and heavily (C) damaged crystals. Scale bars: (B) 200 µm; (C) 700 µm.

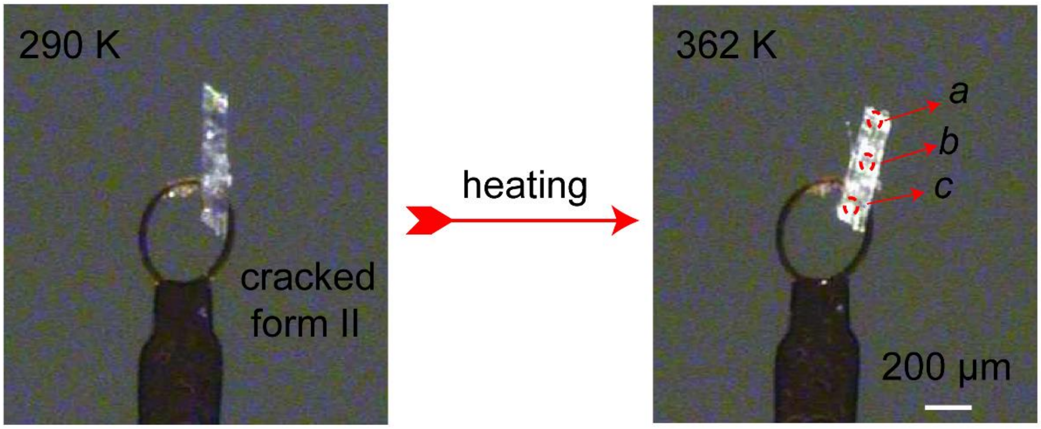

Supplementary Figure 14 | Image of a crystal of TA used in the unit cell measurements of the self-restoration effect. Initially, a crystal of form II was damaged by applying pressure on its (010) face with a metal object. The damaged crystal was taken over the phase transition temperature and its unit cell was determined at three locations (marked as a, b and c). The unit cell corresponds to that of form I. The unit cell details are available from Supplementary Table 3.

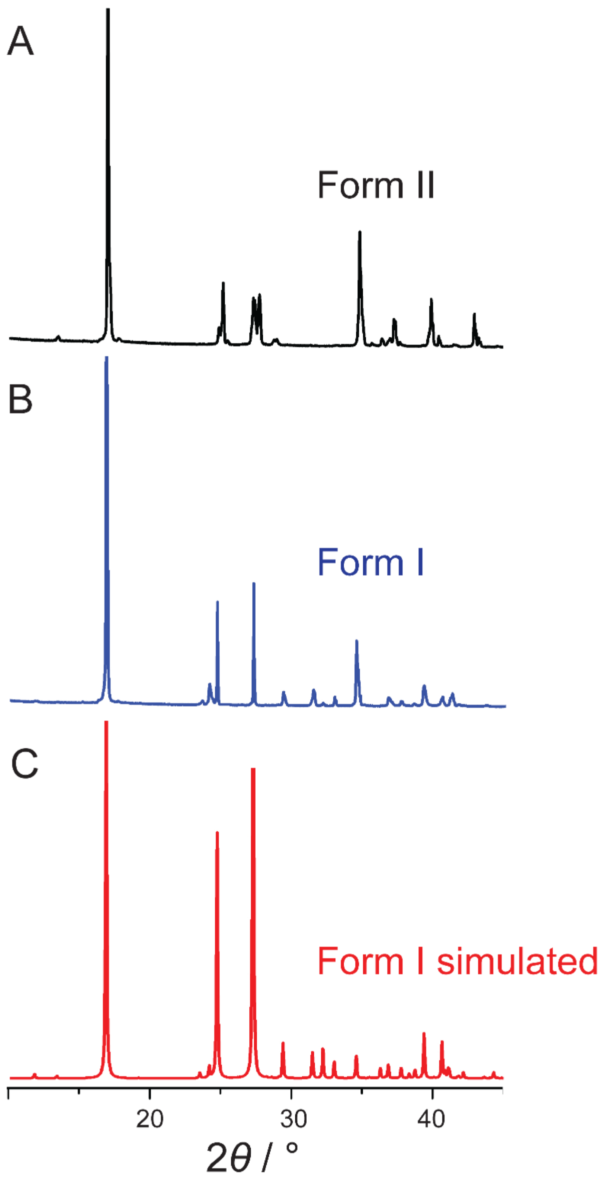

Supplementary Figure 15│ Identification of the high-temperature phase of terephthalic acid by using powder X-ray diffraction. Comparison between the experimental powder X-ray diffraction patterns of form II (A) and form I (B) with the pattern of form I calculated from the crystal structure (C).

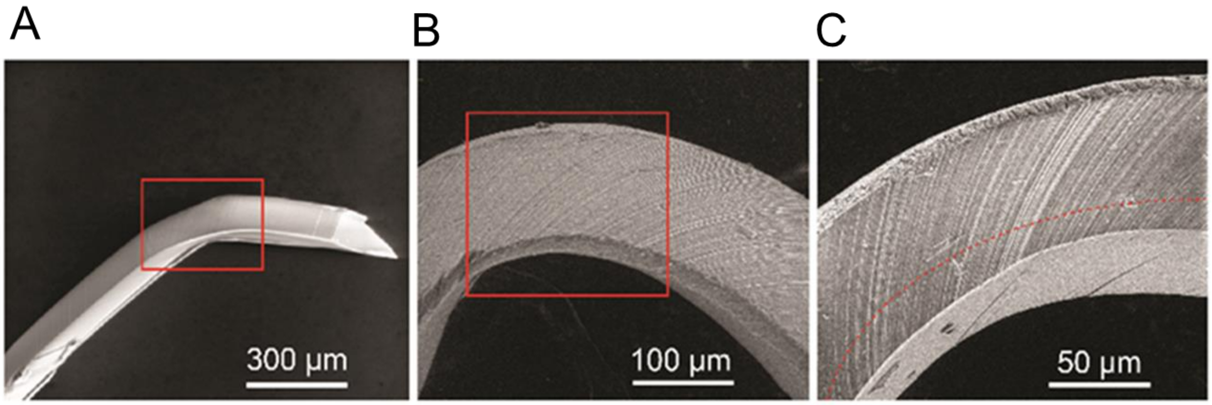

Supplementary Figure 16│Scanning electron microscopy (SEM) images of a surface of a bent crystal of form II terephthalic acid. Panels A, B and C show progressively magnified view of the surface. Striations that appear at approximately 130º in respect to the long crystal axis are clearly visible on both crystal faces. The habit plane is marked with broken red line in panel C.

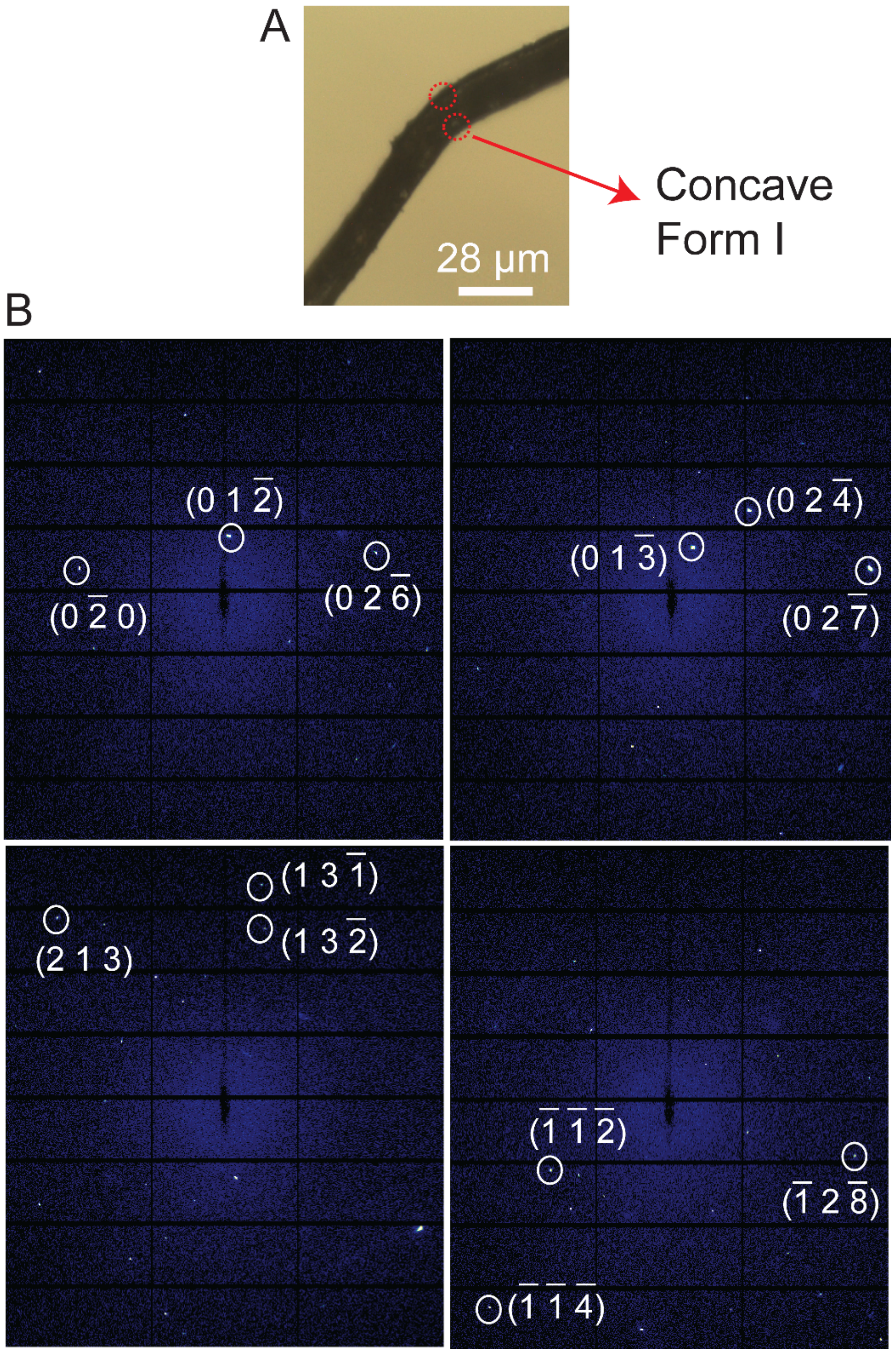

Supplementary Figure 17 | Examples of diffraction images recorded from the concave (inward) side of the bent region of a TA crystal using microfocus X-ray diffraction with synchrotron radiation. The location where the diffraction was recorded is marked with an arrow in panel A, and exemplary diffraction images are shown in panel B.

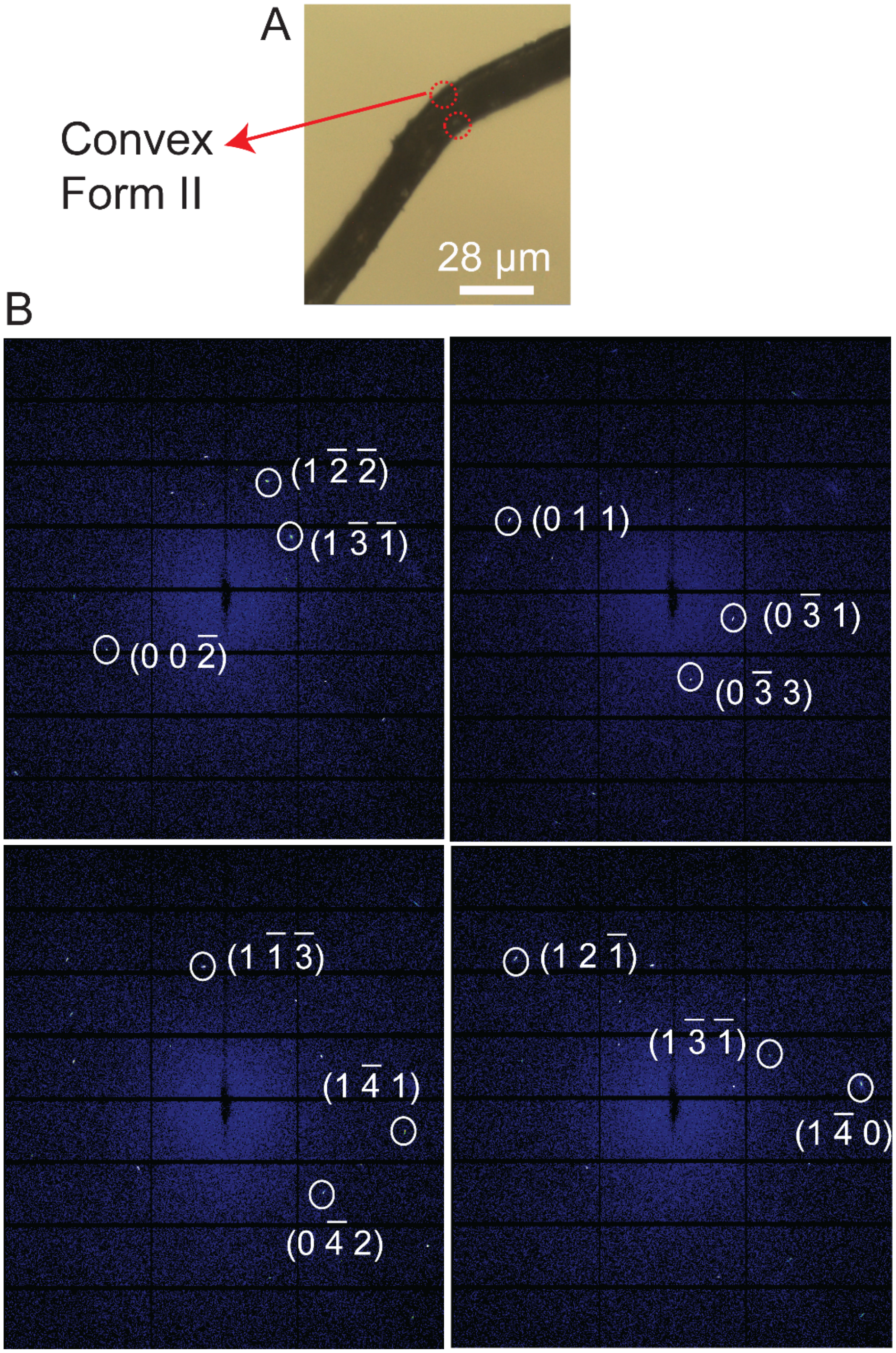

Supplementary Figure 18│Examples of diffraction images recorded from the convex (outward) side of the bent region of terephthalic acid crystal using microfocus X-ray diffraction with synchrotron radiation. The location where the diffraction was recorded is marked with an arrow in panel A and exemplary diffraction images are shown in panel B.

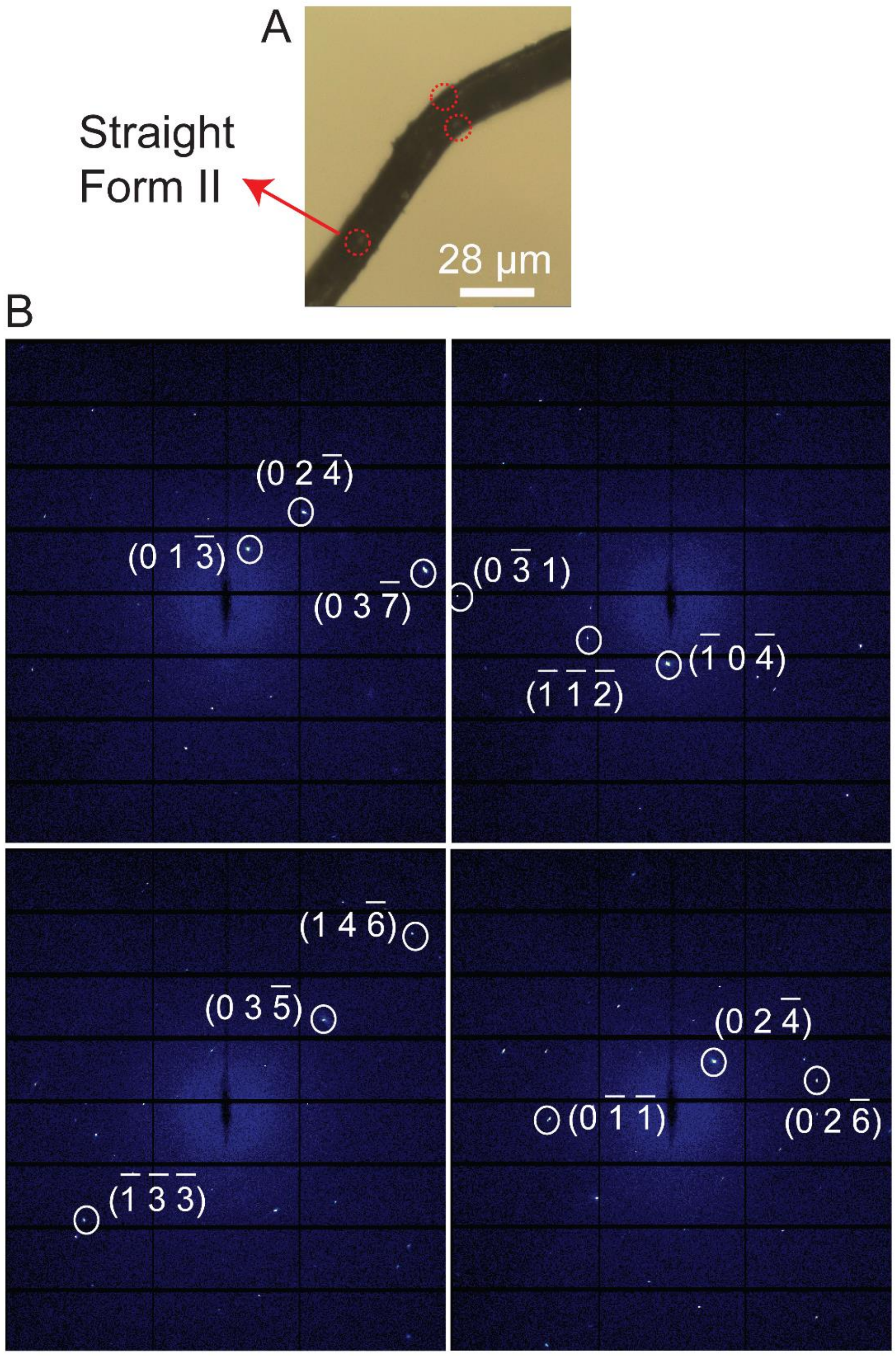

Supplementary Figure 19│Examples of diffraction images recorded from the straight part of a bent crystal of terephthalic acid using microfocus X-ray diffraction with synchrotron radiation. The location where the diffraction was recorded is marked with an arrow in panel A, and exemplary diffraction images are shown in panel B.

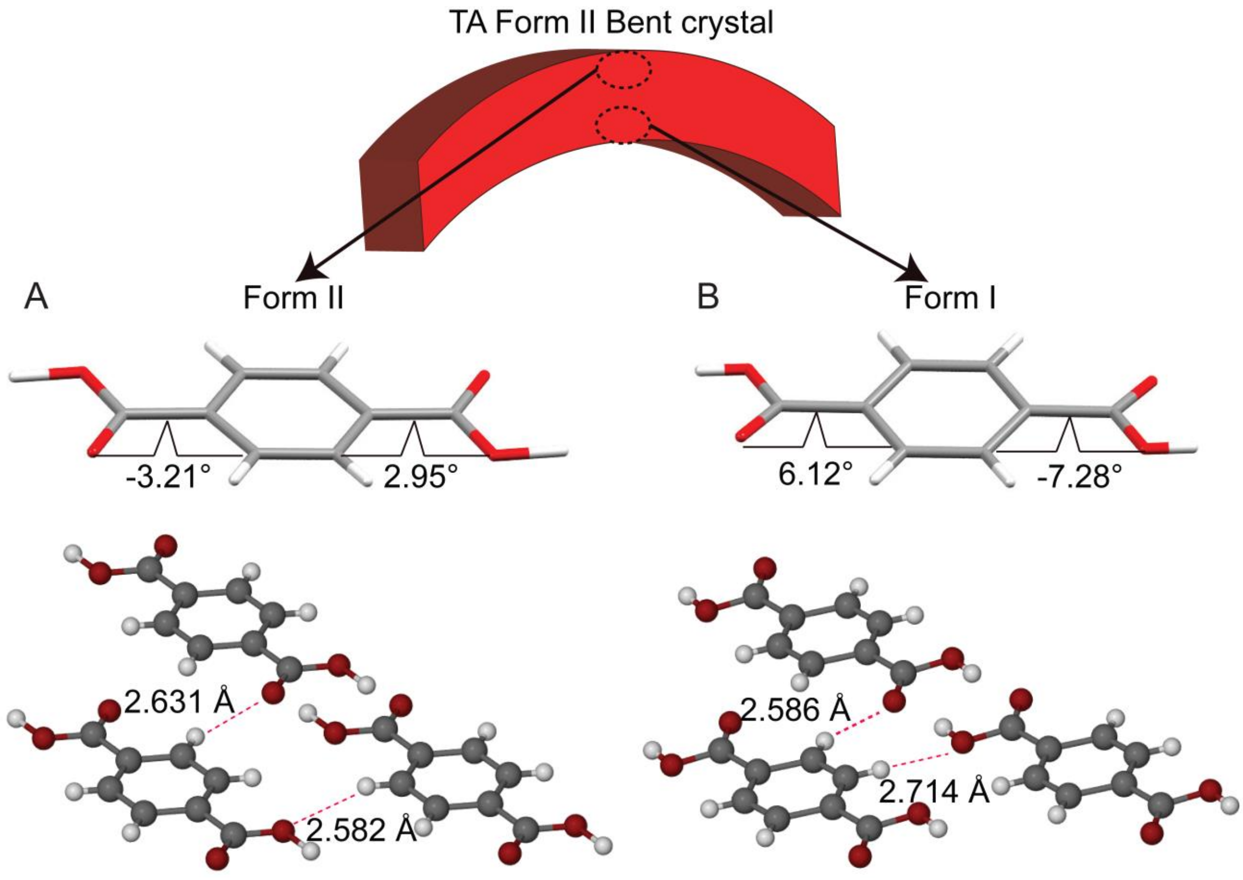

Supplementary Figure 20│Molecular structures and selected geometric features of the molecular packing and intermolecular interactions in forms I (A) and II (B) of terephthalic acid that coexist in the bent region of the crystal.

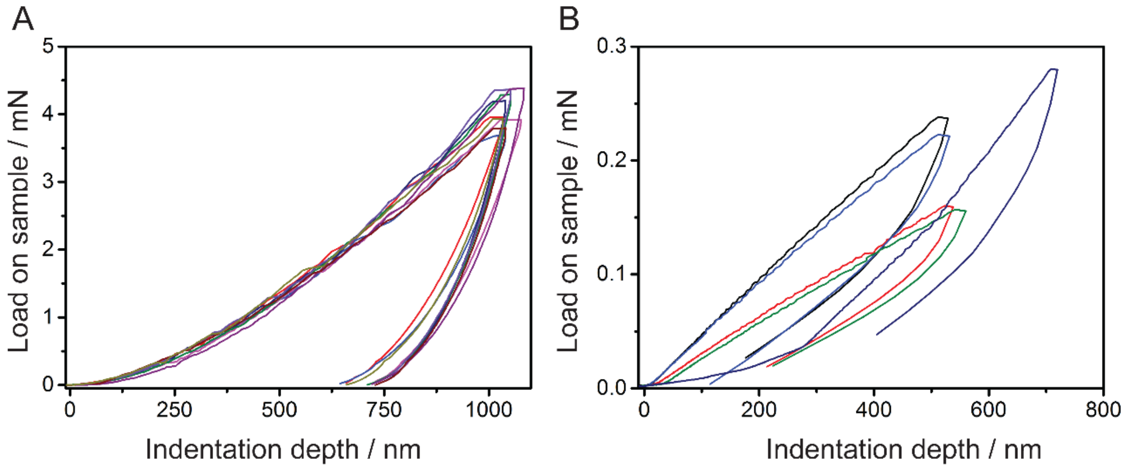

Supplementary Figure 21│ Load-displacement curves of terephthalic acid crystals obtained by nanoindentation on the (010) face of form II (A) and form I (B).

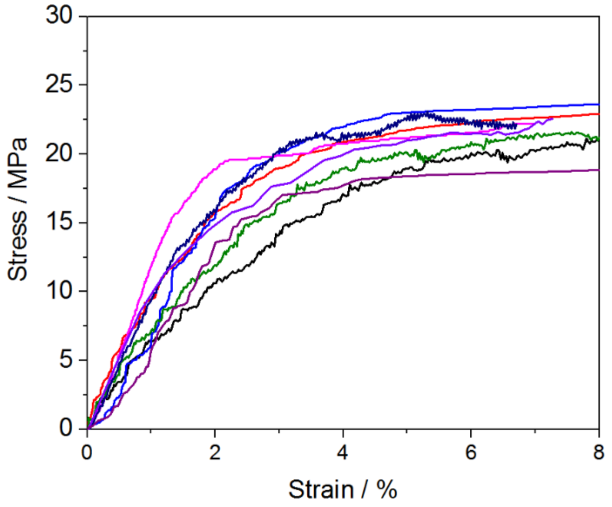

Supplementary Figure 22 | Stress-strain curves for TA crystals of form II bent by the three-point bending method. The critical strain was found to be 2.5 ± 0.2%.

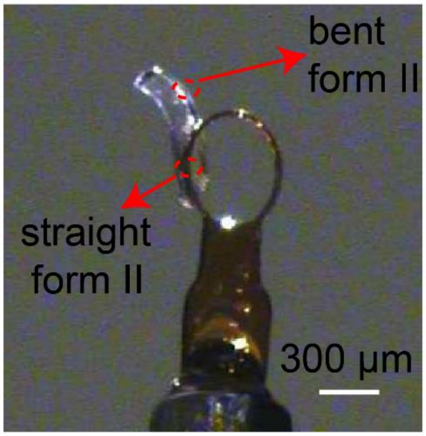

Supplementary Figure 23 | Image of the bent form II crystal of TA used for unit cell measurements on both bent and straight parts. The unit cell details are available from Supplementary Table 5.

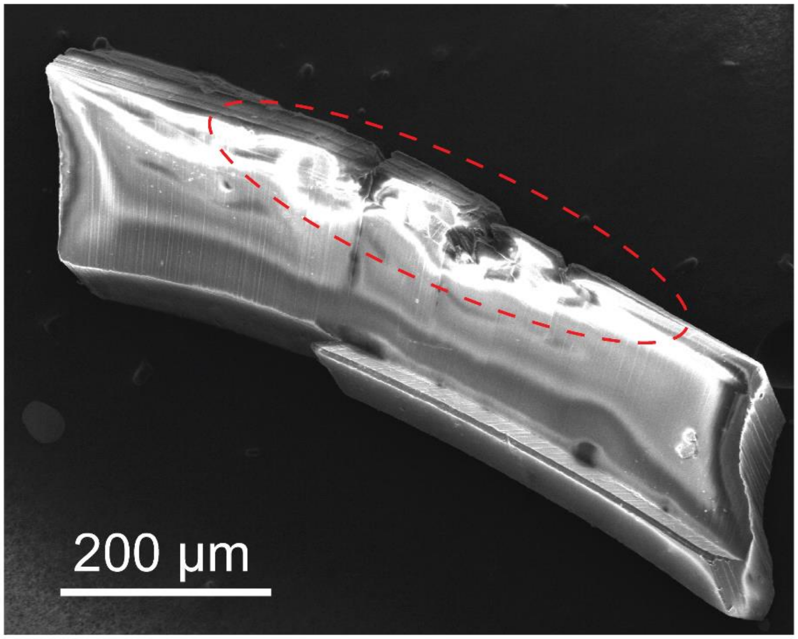

Supplementary Figure 24 | SEM image of a mechanically damaged form II crystal of TA.

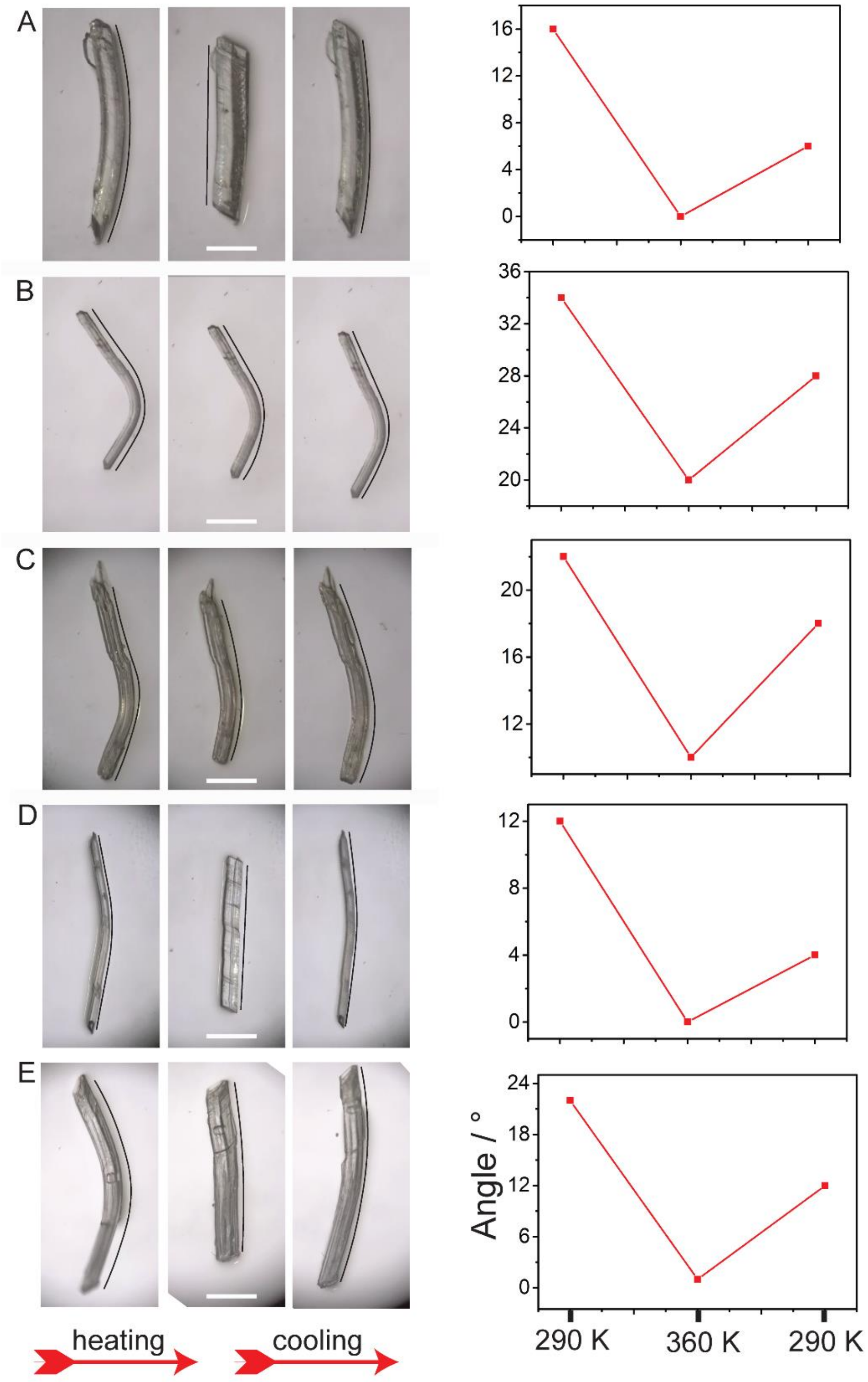

Supplementary Figure 25 | Shape memory effects of crystals of form II terephthalic acid. These bent crystals were then taken over the phase transition to form I by heating from 290 K to 360 K and cooled to 290 K. Scale bars: (A) 600 µm, (B) 800 µm, (C) 500 µm, (D) 700 µm, (E) 900 µm.

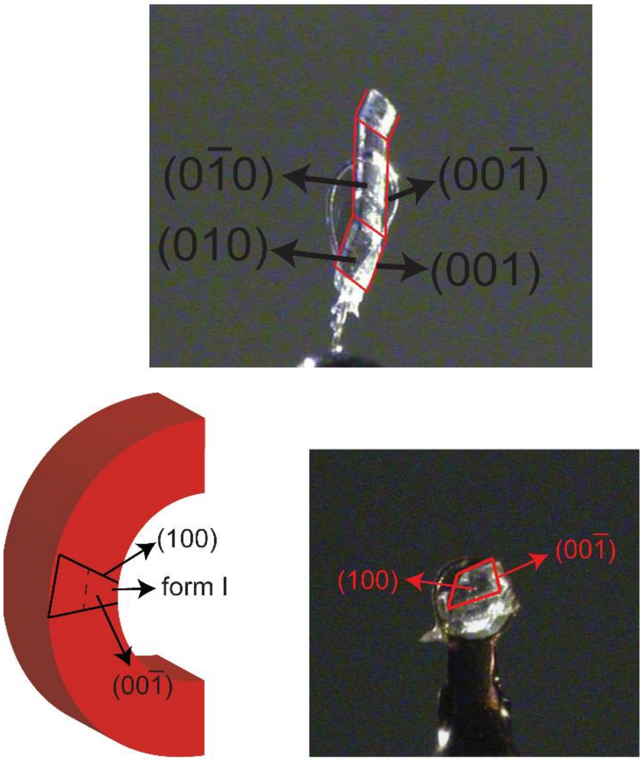

Supplementary Figure 26 | Face indexing of partially converted form II crystal.

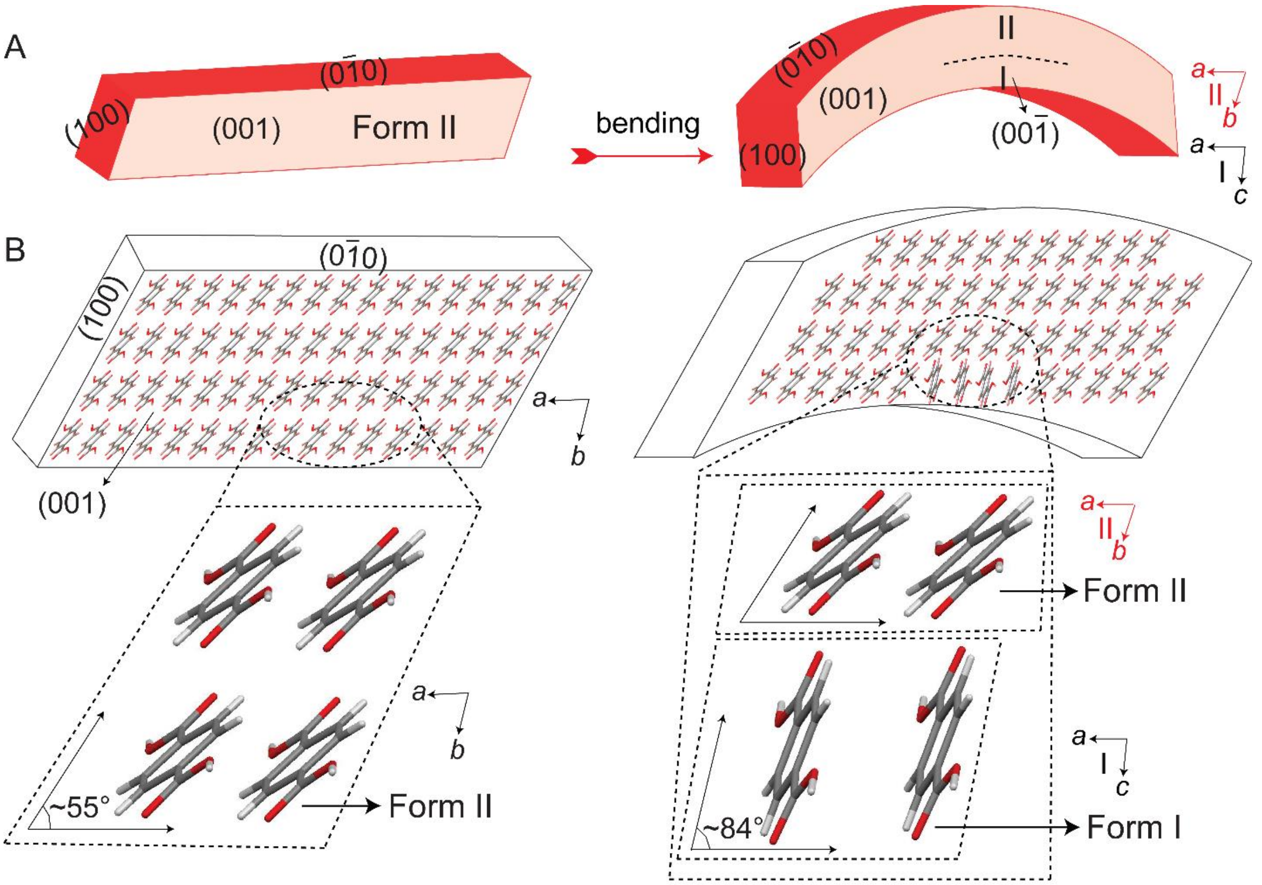

Supplementary Figure 27 | Structural relationship between the two phases in the bent section of the crystal.
