# Supplementary material for: Shape-memory effects in molecular crystals
Source: Nat Commun. 2019 Aug 19;10:3723. doi: 10.1038/s41467-019-11612-z (PMC6700106; doi:10.1038/s41467-019-11612-z)
Supplement: Supplementary file 3 — Description of Additional Supplementary Files [file 41467_2019_11612_MOESM3_ESM.pdf]

Description of Additional Supplementary Files

File name: Supplementary Movie 1

Description: High-speed recording of the mechanosalient effect induced by poking of a crystal of form I which has remained untransformed after cooling to room temperature (frame rate: 1500 s^{-1}; example 1).

File name: Supplementary Movie 2

Description: High-speed recording of the mechanosalient effect induced by poking of a crystal of form I which has remained untransformed after cooling to room temperature (frame rate: 1500 s^{-1}; example 2).

File name: Supplementary Movie 3

Description: High-speed recording of the mechanosalient effect induced by poking of a crystal of form I which has remained untransformed after cooling to room temperature (frame rate: 1500 s^{-1}; example 3).

File name: Supplementary Movie 4

Description: High-speed recording of the mechanosalient effect induced by poking of a crystal of form I which has remained untransformed after cooling to room temperature (frame rate: 1500 s^{-1}; example 4).

File name: Supplementary Movie 5

Description: High-speed recording of the mechanosalient effect induced by poking of a crystal of form I which has remained untransformed after cooling to room temperature (frame rate: 1500 s^{-1}; example 5).

File name: Supplementary Movie 6

Description: Video recording of the procedure used for three-point bending of a crystal of form II at room temperature.

File name: Supplementary Movie 7

Description: A bent crystal of form II is transformed to form I by heating over the phase transition temperature (frame rate: 500 s^{-1}).

File name: Supplementary Movie 8

Description: Dependence of bending angle on the shape restoration. Form II crystal is bent to an angle of 20° and transformed to form I by heating over the phase transition temperature.

File name: Supplementary Movie 9

Description: Dependence of bending angle on the shape restoration. Form II crystal is bent to an angle of 30° and transformed to form I by heating over the phase transition temperature.

File name: Supplementary Movie 10

Description: Dependence of bending angle on the shape restoration. Form II crystal is bent to an angle of 55° and transformed to form I by heating over the phase transition temperature.

File name: Supplementary Movie 11

Description: Dependence of bending angle on the shape restoration. Form II crystal is bent to an angle of 65° and transformed to form I by heating over the phase transition temperature.

File name: Supplementary Movie 12

Description: Video recording of reversible transformation between bent and straight shapes during heating and cooling.

File name: Supplementary Movie 13

Description: Bending of form II crystal by applying pressure on its (010) and $(0\bar{1}0)$ faces and shape memory behavior.

File name: Supplementary Movie 14

Description: Self-restorative effects observed with crystal of form II that was heavily damaged (frame rate: 500 s^{-1}; example 1).

File name: Supplementary Movie 15

Description: Restorative effects in crystal of form II which was damaged by applying pressure on its (001) face by pressing with a metal plate (frame rate: 500 s^{-1}; example 1).

File name: Supplementary Movie 16

Description: Restorative effects in crystal of form II which was damaged by applying pressure on its (001) face by pressing with a metal plate (example 2).

File name: Supplementary Movie 17

Description: Restorative effects in crystal of form II which was damaged by applying pressure on its (001) face by pressing with a metal plate (example 3).

File name: Supplementary Movie 18

Description: Self-restorative effects observed with crystal of form II that was heavily damaged (example 2).

File name: Supplementary Movie 19

Description: Self-restorative effects observed with crystal of form II that was heavily damaged (example 3).
